# Predictors of Outcomes and a Weighted Mortality Score for Moderate to Severe Subdural Hematoma

**DOI:** 10.3390/life14081049

**Published:** 2024-08-22

**Authors:** Sima Vazquez, Aarti K. Jain, Bridget Nolan, Eris Spirollari, Kevin Clare, Anish Thomas, Sauson Soldozy, Syed Ali, Vishad Sukul, Jon Rosenberg, Stephan Mayer, Rakesh Khatri, Brian T. Jankowitz, Justin Singer, Chirag Gandhi, Fawaz Al-Mufti

**Affiliations:** 1 School of Medicine, New York Medical College, Valhalla, NY 10595, USA; ajain10@student.nymc.edu (A.K.J.); fawaz.al-mufti@wmchealth.org (F.A.-M.); 2 Department of Neurosurgery, Westchester Medical Center, Valhalla, NY 10595, USA; 3 Department of Neurology, Westchester Medical Center, Valhalla, NY 10595, USAstephan.mayer@wmchealth.org (S.M.); 4 Department of Neurology, Texas Tech University Health Sciences Center El Paso, Paul L. Foster School of Medicine, El Paso, TX 79409, USA; 5 Department of Neurosurgery, Hospital of the University of Pennsylvania, Philadelphia, PA 19104, USA; 6 Department of Neurological Surgery, Spectrum Health, Grand Rapids, MI 49503, USA

**Keywords:** subdural, subdural hematoma, mortality, mortality scale

## Abstract

As the incidence of subdural hematoma is increasing, it is important to understand symptomatology and clinical variables associated with treatment outcomes and mortality in this population; patients with subdural hematoma were selected from the National Inpatient Sample (NIS) Database between 2016 and 2020 using International Classification of Disease 10th Edition (ICD10) codes. Moderate-to-severe subdural hematoma patients were identified using the Glasgow Coma Scale (GCS). Multivariate regression was first used to identify predictors of in-hospital mortality and then beta coefficients were used to create a weighted mortality score. Of 29,915 patients admitted with moderate-to-severe subdural hematomas, 12,135 (40.6%) died within the same hospital admission. In a multivariate model of relevant demographic and clinical covariates, age greater than 70, diabetes mellitus, mechanical ventilation, hydrocephalus, and herniation were independent predictors of mortality (*p* < 0.001 for all). Age greater than 70, diabetes mellitus, mechanical ventilation, hydrocephalus, and herniation were assigned a “1” in a weighted mortality score. The ROC curve for our model showed an area under the curve of 0.64. Age greater than 70, diabetes mellitus, mechanical ventilation, hydrocephalus, and herniation were predictive of mortality. We created the first clinically relevant weighted mortality score that can be used to stratify risk, guide prognosis, and inform family discussions.

## 1. Introduction

Subdural hematoma has become an increasingly common neurosurgical disease, especially as we experience a shift towards an aging population [1,2]. In addition, many aging patients are increasingly being placed on antiplatelet or anticoagulant medications, portending higher risk of hemorrhage. A recent study found that the overall incidence of chronic subdural hematoma doubled over a 16-year period from 8.2 to 17.6 per 100,000 individuals per year [3]. As the incidence rate of subdural hematoma is increasing, it is important to understand the symptomatology and clinical variables associated with treatment outcomes and mortality in this population. Subdural hematoma is a widely heterogenous and dynamic disease process. Chronic subdural hematoma is defined as a hematoma with a duration greater than three weeks, as opposed to acute subdural hematomas which have a duration of three days up to three weeks [4]. Typically, the pathology results from the tearing of bridging veins, usually in the setting of falls or other minor head trauma [1,2]. However, in 25–50% of all cases, no history of cranial trauma is reported [5]. Expansion of the initial hematoma presentation can occur gradually, leading to an increase in disease severity and often with an insidious clinical presentation mistaken for other neurologic entities [6]. Chronic subdural hematomas may result from the remnants of the acute trauma, as a mixture of clotted blood and serous fluid fill in the subdural space [7]. Leakage of blood and fluid over time leads to growth of the chronic subdural until it overloads local compensatory mechanisms and becomes symptomatic [2]. There is a wide range of symptoms, ranging from behavioral changes and seizures to hemiparesis and headaches [7].

In the past, the severity of chronic subdural hematomas has been based off clinical presentation. According to the Markwalder grading system of chronic subdural hematoma severity, chronic subdural hematomas are assessed on a scale from 0–4 [8]. Moderately severe subdural hematomas present with mild symptoms or variable neurological deficits, such as hemiparesis. The most severe patients are those who show severe focal signs or present as comatose with decerebrate or decorticate posturing [9]. Factors that affect the severity of the hematoma include hematoma volume, speed of volume expansion, location, and presence of mass effect.

The current treatment of chronic subdural hematoma includes medical and surgical management. For asymptomatic patients, conservative measures, such as observation, intracranial pressure control, anticoagulation reversal, and repeated examinations are carried out. Indications for surgery include presence of symptoms, thick hematoma (>10 mm), midline shift greater than 7 mm, and radiographic progression after conservative management. The main surgical modalities are burr hole or craniotomy evacuation. More recently, middle meningeal artery (MMA) embolization has become another method for the surgical management of chronic subdural hematoma. Ongoing clinical trials are assessing the efficacy and safety of usage of MMA embolization for treatment of chronic subdural hematomas.

Clinical outcomes of subdural hematoma may vary widely as data are limited to retrospective studies, with mortality rates reported from 0% to over 30% [9] and morbidity rates ranging from 0% to 25% [2,9]. This wide range is likely due to the heterogenous nature of subdural hematomas, the large spectrum of disease presentation and severity, and the treatment modalities.

Prognostic factors, such as increased age, obesity, low Glasgow Coma Scale (GCS), presence of medical comorbidities, and coagulopathy, have been linked to increased morbidity and mortality rates in individuals with chronic subdural hematoma [10]. Male sex is another factor tied to increased risk for chronic subdural hematoma, though an association with mortality has not been explored [9]. Midline shifts greater than 10 mm and hematoma thickness over 30 mm have been linked with worse outcomes [9,11]. Mortality scores have been devised for hematomas in specific populations, such as the elderly and postoperative patients [2]. However, to date, there is no scoring system available for clinicians that can be used to help predict outcomes in all subdural hematomas, regardless of time course.

Utilizing the National Inpatient Sample (NIS), we conducted a retrospective survey examining predictors of mortality in patients with moderate-to-severe subdural hematomas. In addition, we present a clinically relevant weighted mortality score for patients with subdural hematomas.

## 2. Materials and Methods

### 2.1. Data Source and Patient Selection

The National Inpatient Sample (NIS), which is managed by the Healthcare Cost and Utilization Project (HCUP), is recognized as the most expansive database of its kind, featuring an extensive collection of inpatient hospital admission records from all over the United States [HCUP National Inpatient Sample (NIS). Healthcare Cost and Utilization Project (HCUP). 2010–2019. Agency for Healthcare Research and Quality, Rockville, MD. www.hcup-us.ahrq.gov/kidoverview.jsp (accessed on 23 November 2023)]. In our detailed study, the NIS database was thoroughly examined for the period from 2016 to 2020, with a specific focus on patients diagnosed primarily with subdural hematoma. This particular diagnosis was identified using designated codes from the International Classification of Diseases, Tenth Revision (ICD10), specifically codes I62 and S06.5X0. These codes include patients admitted with “nontraumatic acute subdural hemorrhage”, “nontraumatic subacute subdural hemorrhage”, “nontraumatic chronic subdural hemorrhage”, and “traumatic subdural hemorrhage”. Patients who were found to have a Glasgow Coma Scale (GCS) score of less than 12 were categorized as suffering from moderate-to-severe conditions. The primary objective of this study was to scrutinize and compare the outcomes of these moderate-to-severe subdural hematoma cases, focusing on the distinctions between patients who died during their hospitalization and those who were discharged alive.

### 2.2. Data Items

The initial phase of our analysis involved a meticulous comparison of baseline demographic characteristics such as age, gender, race/ethnicity, and insurance coverage, along with clinical comorbidities including anticoagulation status, diabetes mellitus, hyperlipidemia, hypertension, and obesity between those who deceased and those who were discharged. Additional clinical parameters such as the incidences of hyponatremia, cardiac arrest, and non-epileptic seizures were carefully scrutinized for both groups. This was followed by a deeper exploration into clinical severity factors, encompassing the necessity for mechanical ventilation, the presence of hydrocephalus, cerebral herniation, hemiplegia, and aphasia. Moreover, the study extensively evaluated the performance of various medical procedures among the patients, which included interventions such as middle meningeal artery (MMA) embolization, craniotomy, and the use of a burr hole or subdural evacuating port system (SEPS).

### 2.3. Statistical Analysis

In the statistical analysis segment of our research, categorical variables were methodically compared using Pearson’s chi-squared test, while the distribution normality of continuous variables was assessed through the Kolmogorov–Smirnov test. Based on the results concerning distribution normality, continuous variables were analyzed either using the Student’s *t*-test or the Mann–Whitney U test. In addition, an exhaustive multivariate regression analysis was conducted to identify the independent demographic and clinical predictors of mortality in patients with moderate-to-severe subdural hematoma. These models assumed a representative sample and independent variables were below collinearity thresholds. The statistically significant variables that were uncovered were then integrated as covariates into a detailed multivariable logistic regression model, which was instrumental in developing a weighted mortality score based on the derived beta coefficients. This mortality score was subsequently validated by evaluating the mortality rates across various score values within a carefully selected cohort of moderate-to-severe subdural hematoma patients from the year 2019. All statistical analyses were executed using the Statistical Product and Service Solutions (SPSS) version 29 software, with a set threshold for statistical significance at *p* < 0.05 (IBM Corp. Released 2020. IBM SPSS Statistics for Windows, Version 28.0. Armonk, NY, USA: IBM Corp.).

### 2.4. Data Availability

The comprehensive data supporting this investigation are available for scholarly review upon a structured request to the corresponding author. These requests are required to comply with the strict onboarding procedures as stipulated by the Healthcare Cost and Utilization Project, which are designed to ensure adherence to data privacy and usage policies. The availability of these data promotes further research and enables the validation and replication of our study’s findings by the wider scientific and academic community.

## 3. Results

### 3.1. Cohort Characteristics

From 2016 to 2020, a total of 740,935 hospital admissions were recorded with the principal diagnosis of subdural hematoma. The overall mortality rate for these cases was calculated at 9.3%, amounting to 69,125 deaths. Specifically, 29,915 patients (representing 43.3% of the total cases) were admitted with moderate-to-severe subdural hematoma. Of these, 12,135 (40.6% of the total cases) died during the same hospital admission. Analyzing the demographics and conditions of those who died inpatient, we found significant associations on chi-squared analysis: individuals older than 70 years had a higher likelihood of mortality (OR 2.16, 95% CI 2.06–2.27), as did female patients (OR 1.12, 95% CI 1.07–1.17 *p* < 0.001), those of Caucasian race (OR 1.18, 95% CI 1.12–1.23 *p* < 0.001), and patients covered by public insurance (OR 1.27, 95% CI 1.21–1.34 *p* < 0.001), compared to those who survived and were discharged. Moreover, conditions such as long-term anticoagulation therapy (OR 2.30, 95% CI 2.14–2.46 *p* < 0.001), diabetes mellitus (OR 1.52, 95% CI 1.42–1.62 *p* < 0.001), hyperlipidemia (OR 1.14, 95% CI 1.07–1.22 *p* < 0.001), hypertension (OR 1.06, 95% CI 1.01–1.11 *p* < 0.001), and obesity (OR 1.20, 95% CI 1.04–1.38 *p* < 0.001) were more prevalent among patients who died than those discharged (Table 1).

On chi-squared analysis, patients who deceased demonstrated significantly lower rates of hyponatremia (OR 0.44, 95% CI 0.40–0.48 *p* < 0.001) and seizures (OR 0.48, 95% CI 0.44–0.53 *p* < 0.001), but higher instances of cardiac arrest (OR 12.96, 95% CI 10.82–15.57 *p* < 0.001) during their hospital stay compared to those who survived (Table 1). Additional complications such as the need for mechanical ventilation (OR 1.32, 95% CI 1.26–1.38 *p* < 0.001), hydrocephalus (OR 1.48, 95% CI 1.28–1.71 *p* < 0.001), and brain herniation (OR 2.83, 95% CI 2.68–2.99 *p* < 0.001) were more commonly seen in patients who died inpatient than those discharged. Conversely, occurrences of hemiplegia (OR 0.44, 95% CI 0.41–0.49 *p* < 0.001) and aphasia (OR 0.24, 95% CI 0.21–0.27 *p* < 0.001) were less frequent in deceased patients compared to those who survived (Table 1).

Chi-squared analysis showed that less frequent medical interventions in deceased patients included external ventricular drain (EVD) placement (OR 0.81, 95% CI 0.74–0.88 *p* < 0.001), procedures involving burr hole or subdural evacuating port system (SEPS) (OR 0.46, 95% CI 0.40–0.52 *p* < 0.001), craniotomy (OR 0.88, 95% CI 0.76–1.01 *p* = 0.037), and middle meningeal artery (MMA) embolization (OR 0.21, 95% CI 0.08–0.53 *p* < 0.001), all suggesting a lower rate of invasive intervention among those who did not survive their hospital stay.

### 3.2. Predictors of Mortality in Patients with Subdural Hematoma

Significant independent predictors of mortality on multivariate regression included age over 70 (OR 2.24, 95% CI 2.12–2.36 *p* < 0.001) (Table 2), demonstrating a strong correlation between advanced age and increased mortality risk. Gender and insurance status also played a discernible role, with female patients showing slightly lower odds of mortality (OR 0.95, 95% CI 0.90–0.99 *p* < 0.032) and those with public insurance displaying a reduced mortality risk (OR 0.92, 95% CI 0.88–0.98 *p* < 0.001).

Moreover, multivariate regression using clinical covariates showed that long-term anticoagulation therapy (OR 2.08, 95% CI 1.97–2.20 *p* < 0.001), diabetes mellitus (OR 1.66, 95% CI 1.54–1.79 *p* < 0.001), the requirement for mechanical ventilation (OR 1.44, 95% CI 1.37–1.52 *p* < 0.001), hydrocephalus (OR 1.28, 95% CI 1.09–1.49 *p* < 0.001), and herniation (OR 2.64, 95% CI 2.50–2.80 *p* < 0.001) were also highly predictive of mortality (Table 3).

### 3.3. Moderate-to-Severe Subdural Hematoma Mortality Score

**Figure 1:**
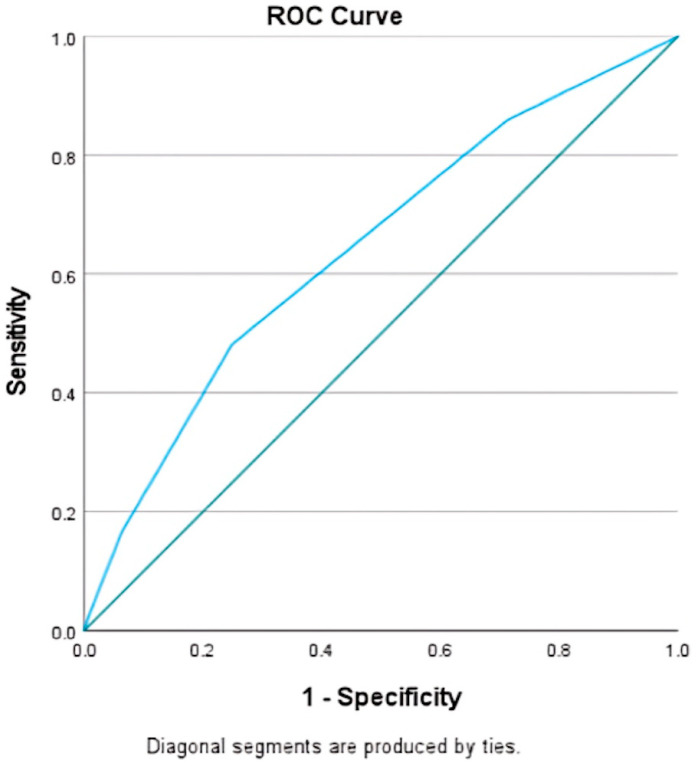
Area under the curve for the weighted mortality score. Area under the curve ROC analysis depicting goodness of fit of mortality score; AUC = 0.64, standard error 0.007. Green line is reference line.

Utilizing logistic regression models, a detailed mortality outcome stratification scale was developed. This scale quantitatively assessed risk factors including age over 70 (OR 2.32, 95% CI 2.19–2.47 *p* < 0.001), diabetes mellitus (OR 1.26, 95% CI 1.17–1.35 *p* < 0.001), mechanical ventilation (OR 1.44, 95% CI 1.37–1.51 *p* < 0.001), hydrocephalus (OR 1.27, 95% CI 1.09–1.49 *p* < 0.001), and herniation (OR 2.69, 95% CI 2.54–2.85 *p* < 0.001), and each variable was assigned “1” in the model coefficient (Table 4 and Table 5). The receiver operating characteristic (ROC) curve demonstrated the model’s efficacy with an area under the curve (AUC) of 0.64 (Figure 1).

**Figure 2:**
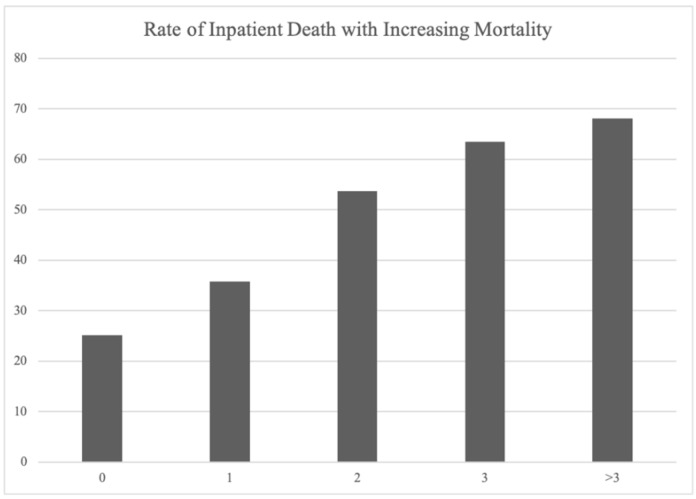
Rate of mortality with increasing mortality score in select cohort. Bar graph depicting rate of mortality with each increase point in mortality score in a select cohort of moderate–severe subdural hematoma patients in 2019.

The subdural hematoma mortality score showed accurate prediction of inpatient mortality when applied to a select cohort of patients with moderate-to-severe chronic subdural hematoma in 2019 (Figure 2). The mortality rate associated with a score of 0 was 25.2%. It was followed by a 35.7% mortality rate associated with a score of 1, 53.7% associated with a score of 2, 63.5% associated with a score of 3, and 68% associated with a score above 3 (Figure 2).

## 4. Discussion

Ultimately, the mortality score was able to distinguish risk factors for inpatient mortality in all subdural hematomas to improve prognosis, regardless of the chronicity of pathology. Patients with moderate-to-severe subdural hematoma who died inpatient were more likely to have been on long-term anticoagulation therapy and experienced more severe hospital courses, including cardiac arrest, mechanical ventilation, and hydrocephalus. They were also more likely to have a cardiac arrest compared to those who were discharged. Patients who died inpatient were less likely to have cortical symptoms of aphasia and hemiplegia but more likely to develop hydrocephalus, herniation, and coma, suggesting a more severe presentation in which focal signs such as aphasia or hemiplegia were masked by global deficits [12]. Patients who were discharged were more likely to have public insurance and experience the clinical characteristics of hyponatremia and seizures. Patients who received surgical treatment in the way of EVD placement, burr hole or subdural evacuating port system (SEPS) placement, craniotomy, and MMA embolization were more likely to be discharged than to die inpatient.

Demographic predictors of mortality were female sex, age over 70, and public insurance. Clinical predictors included long-term anticoagulation, hypertension, hyperlipidemia, and status epilepticus. Caucasian race, Q1 median income, obesity, and EVD placement were not predictors of mortality. When accounting for sample size, the factors that were included in the mortality score were age over 70, diabetes mellitus, mechanical ventilation, hydrocephalus, and herniation. Ultimately, the mortality score predicted inpatient mortality in a select population of patients with subdural hematoma.

There is a dense body of literature discussing predictors of outcomes in patients with nontraumatic subdural hematomas. Other mortality scores have been designed for subdural hematomas in specific populations. For example, the Subdural Hematoma in the Elderly (SHE) score was created to predict 30-day mortality. However, the factors evaluated were only age, GCS, and hematoma volume [13]. It did not take into account anticoagulant use, presence of comorbidities, and other factors that may lead to poor clinical and functional outcomes. More importantly, it was designed to only be applied to the elderly patient population, and therefore cannot be generalized to a larger population. The Oslo CSDH scale, a mortality score for chronic subdural hematoma, only looks to predict recurrence of the hematoma after initial burr-hole surgery. Components of the Oslo CSDH mortality score included density changes in CT scan imaging, preoperative hematoma volume, and postoperative residual cavity volume [14]. As a result, physicians have not had a score to identify mortality risk in all patients with moderate-to-severe subdural hematoma regardless of the cause [12,15,16,17].

Predictors of poor outcomes include low Glasgow Coma Scale (GCS), female sex, older age, and poor admission neurological status [12,15,16,17]. In this nationwide cohort, mortality was predicted by older age (greater than 70) and certain clinical markers of severity (diabetes mellitus, mechanical ventilation, hydrocephalus, and herniation). Individuals with increased age have been shown to present with increased neurological deficits compared to their younger counterparts [18]. Also, presence of pre-existing comorbidities is also correlated to older age [19]. Therefore, many of these factors that correlate to poor outcomes are intrinsically linked. Using these variables, we created the first clinically relevant mortality score that can be used to stratify risk, guide prognosis, and inform family discussions. As seen in a select cohort from 2019, a score above 3 was associated with an almost 70% rate of inpatient death.

Having a mortality score allows for quicker identification of at-risk patients and for application of the proper treatment modalities. Operative or endovascular management was not associated with mortality in our cohort. Operative management appears to be protective in acute or subacute subdural hematoma but may not be associated with mortality in more chronic subdural hematomas [5]. In older populations, craniotomy was associated with hospital mortality [6]. Identifying specific populations that may benefit from certain treatments is an important area of further study. Furthermore, it is of interest to continue following the mortality trend as rates of middle meningeal embolization for chronic subdural hematoma rise across the nation [20,21]. Moreover, there is an incidence rate (3 to 20%) of recurrence of chronic subdural hematomas [22]. Designing predictive scores based on prognostic factors for recurrence can help to identify which patients require increased monitoring after initial recovery.

This study is limited by its retrospective nature, as it uses the NIS database. The NIS lacks objective measures such as timing and chronicity, size and location of the subdural hematoma, neurologic exam, and lab values. Measurements, such as midline shift and hematoma thickness, could not be factored into this mortality scoring system due to a lack of available data from the NIS database. However, this allowed us to focus on clinical variables to create a score based on presentation independent of these objective measures. We used ICD codes consistent with those used in the literature, but heterogeneity in ICD coding throughout the nation may still lead to misclassification [9]. Our mortality score applies to a wide nationwide cohort; further studies are warranted to identify factors that should be given more or less weight in certain populations. Lastly, our study focused on risk factors for both acute and chronic subdural hematomas, which have differing etiologies, age groups, and treatment protocols. Future studies can look to stratifying between acute and chronic subdural hematomas in order to identify risk factors for inpatient mortality in each group.

## 5. Conclusions

Age greater than 70, diabetes mellitus, mechanical ventilation, hydrocephalus, and brain herniation were all found to be significant predictors of mortality in patients diagnosed with moderate-to-severe subdural hematoma. Based on these critical variables, we lay the foundation for the development of an innovative, clinically relevant weighted mortality score aimed at stratifying risk levels and guiding discussions of prognosis.

## Figures and Tables

**Table 1:** Inpatient death in moderate–severe subdural hematoma patients.

	Total (29,915)	Discharged (17,780, 59.4%)	Inpatient Death (12,135, 40.6%)	OR (95% CI)	*p* Value
Demographics					
Age > 70	10,860 (36.3%)	5165 (29%)	5695 (46.9%)	2.16 (2.06–2.27)	<0.001
Female	10,550 (35.3%)	6085 (34.2%)	4465 (36.8%)	1.12 (1.07–1.17)	<0.001
Caucasian	17,735 (59.3%)	10,260 (57.7%)	7475 (61.6%)	1.18 (1.12–1.23)	<0.001
Public Insurance	19,125 (63.9%)	10,970 (61.7%)	8155 (67.2%)	1.27 (1.21–1.34)	<0.001
Clinical Comorbidities					
Long-term Anticoagulation	3630 (12.1%)	1505 (8.5%)	2125 (17.5%)	2.30 (2.14–2.46)	<0.001
Diabetes mellitus	4475 (15%)	2270 (12.8%)	2205 (18.2%)	1.52 (1.42–1.62)	<0.001
Hyperlipidemia	4655 (15.6%)	2640 (14.8%)	2015 (16.6%)	1.14 (1.07–1.22)	<0.001
Hypertension	10,630 (35.5%)	6220 (35%)	4410 (36.3%)	1.06 (1.01–1.11)	<0.001
Obesity	825 (2.8%)	455 (2.6%)	370 (3%)	1.20 (1.04–1.38)	<0.001
Clinical Characteristics					
Hyponatremia	3255 (10.9%)	2460 (13.8%)	795 (6.6%)	0.44 (0.40–0.48)	<0.001
Cardiac Arrest	1230 (4.1%)	135 (0.8%)	1095 (9%)	12.96 (10.82–15.57)	<0.001
Seizures	2295 (7.7%)	1705 (9.6%)	590 (4.9%)	0.48 (0.44–0.53)	<0.001
Clinical Severity Variables					
Mechanical Ventilation	13,935 (46.6%)	7785 (43.8%)	6150 (50.7%)	1.32 (1.26–1.38)	<0.001
Hydrocephalus	740 (2.5%)	370 (2.1%)	370 (3%)	1.48 (1.28–1.71)	<0.001
Herniation	6955 (23.2%)	2780 (15.6%)	4175 (34.4%)	2.83 (2.68–2.99)	<0.001
Hemiplegia	2700 (9%)	2040 (11.5%)	660 (5.4%)	0.44 (0.41–0.49)	<0.001
Aphasia	1670 (5.6%)	1425 (8%)	245 (2%)	0.24 (0.21–0.27)	<0.001

Chi-squared analysis comparing demographics, comorbidities, clinical characteristics, clinical severity, and procedures between patients who died and those who were discharged.

**Table 2:** Demographic predictors of mortality in moderate–severe subdural hematoma.

Variable	OR (95% CI)	*p* Value
Female	0.95 (0.90–0.99)	0.032
Age > 70	2.24 (2.12–2.36)	<0.001
Caucasian	1.02 (0.97–1.07)	0.387
Public Insurance	0.92 (0.88–0.98)	<0.001
Q1 Median Income	0.96 (0.91–1.01)	0.114

Multivariate regression of demographic variables predictive of mortality in moderate–severe subdural hematoma.

**Table 3:** Clinical predictors of mortality in moderate–severe subdural hematoma.

Variable	OR (95% CI)	*p* Value
Long-term anticoagulation	2.08 (1.97–2.20)	<0.001
Diabetes Mellitus	1.66 (1.54–1.79)	<0.001
Hyperlipidemia	0.81 (0.75–0.87)	<0.001
Hypertension	0.84 (0.80–0.89)	<0.001
Obesity	1.12 (0.97–1.30)	0.137
Mechanical Ventilation	1.44 (1.37–1.52)	<0.001
Hydrocephalus	1.28 (1.09–1.49)	<0.001
External ventricular drain	0.93 (0.85–1.02)	0.111
Hyponatremia	0.45 (0.41–0.49)	<0.001
Herniation	2.64 (2.50–2.80)	<0.001
Non-epileptic Seizure	0.51 (0.46–0.56)	<0.001
Status Epilepticus	0.78 (0.61–0.99)	0.043

Multivariate regression of clinical variables predictive of mortality in moderate–severe subdural hematoma.

**Table 4:** Combined model predictive of mortality in moderate–severe subdural hematoma.

	B Coefficient	OR (95% CI)	*p* Value	Weighted Coefficient
Female	−0.05	0.90 (0.90–1.00)	0.061	
Age > 70	0.84	2.32 (2.19–2.47)	<0.001	1
Public Insurance	−0.09	0.91 (0.86–0.96)	<0.001	
Long Term Steroids	−0.34	0.71 (0.53–0.95)	0.022	
Diabetes mellitus	0.23	1.26 (1.17–1.35)	<0.001	1
Hyperlipidemia	−0.19	0.82 (0.77–0.89)	<0.001	
Hypertension	−0.18	0.84 (0.80–0.89)	<0.001	
Mechanical Ventilation	0.36	1.44 (1.37–1.51)	<0.001	1
Hydrocephalus	0.24	1.27 (1.09–1.49)	<0.001	1
Hyponatremia	−0.80	0.45 (0.41–0.49)	<0.001	
Herniation	0.99	2.69 (2.54–2.85)	<0.001	1
Seizures	−0.68	0.51 (0.46–0.56)	<0.001	
Status Epilepticus	−0.23	0.79 (0.62–1.01)	0.064	

Combined demographics and clinical variables predictive of mortality in moderate–severe subdural hematoma with their associated weighted coefficients.

**Table 5:** Weighted mortality score for moderate–severe subdural hematoma.

Variable	Coefficient
Age > 70	1
Diabetes mellitus	1
Mechanical Ventilation	1
Hydrocephalus	1
Herniation	1

Variables included in the mortality score and their respective weighted coefficients.

## Data Availability

Data available to be shared upon reasonable request from the corresponding author and proper onboarding procedures as described by HCUP.

## References

[1] Ducruet A.F., Grobelny B.T., Zacharia B.E., Hickman Z.L., DeRosa P.L., Anderson K., Sussman E., Carpenter A., Connolly E.S. (2012). The surgical management of chronic subdural hematoma. Neurosurg. Rev..

[2] Feghali J., Yang W., Huang J. (2020). Updates in Chronic Subdural Hematoma: Epidemiology, Etiology, Pathogenesis, Treatment, and Outcome. World Neurosurg..

[3] Rauhala M., Helén P., Huhtala H., Heikkilä P., Iverson G.L., Niskakangas T., Öhman J., Luoto T.M. (2020). Chronic subdural hematoma—Incidence, complications, and financial impact. Acta Neurochir..

[4] Cenic A., Bhandari M., Reddy K. (2005). Management of chronic subdural hematoma: A national survey and literature review. Can. J. Neurol. Sci. J. Can. Sci. Neurol..

[5] Sastry R.A., Pertsch N., Tang O., Shao B., Toms S.A., Weil R.J. (2020). Frailty and Outcomes after Craniotomy or Craniectomy for Atraumatic Chronic Subdural Hematoma. World Neurosurg..

[6] Weigel R., Schilling L., Krauss J.K. (2022). The pathophysiology of chronic subdural hematoma revisited: Emphasis on aging processes as key factor. GeroScience.

[7] Sahyouni R., Goshtasbi K., Mahmoodi A., Tran D.K., Chen J.W. (2017). Chronic Subdural Hematoma: A Historical and Clinical Perspective. World Neurosurg..

[8] Markwalder T.-M. (2000). The course of chronic subdural hematomas after burr-hole craniostomy with and without closed-system drainage. Neurosurg. Clin. N. Am..

[9] Nouri A., Gondar R., Schaller K., Meling T. (2021). Chronic Subdural Hematoma (cSDH): A review of the current state of the art. Brain Spine.

[10] Kim Y.-I., Lee J.-H., Park S.-W., Nam T.-K., Min B.-K., Hwang S.-N. (2008). Analysis of Prognostic Factors for Chronic Subdural Hematoma. J. Korean Neurotraumatol. Soc..

[11] Kwon C.-S., Al-Awar O., Richards O., Izu A., Lengvenis G. (2018). Predicting Prognosis of Patients with Chronic Subdural Hematoma: A New Scoring System. World Neurosurg..

[12] Rozzelle C.J., Wofford J.L., Branch C.L. (1995). Predictors of hospital mortality in older patients with subdural hematoma. J. Am. Geriatr. Soc..

[13] Alford E.N., Rotman L.E., Erwood M.S., Oster R.A., Davis M.C., Pittman H.B.C., Zeiger H.E., Fisher W.S. (2019). Development of the Subdural Hematoma in the Elderly (SHE) score to predict mortality. J. Neurosurg..

[14] Stanišić M., Pripp A.H. (2017). A Reliable Grading System for Prediction of Chronic Subdural Hematoma Recurrence Requiring Reoperation After Initial Burr-Hole Surgery. Neurosurgery.

[15] Weimer J.M., Gordon E., Frontera J.A. (2017). Predictors of Functional Outcome After Subdural Hematoma: A Prospective Study. Neurocrit. Care.

[16] Schneck M.J., Maheswaran M., Leurgans S. (2004). Predictors of outcomes after nontraumatic subdural hematoma. J. Stroke Cerebrovasc. Dis..

[17] Wang S., Ma Y., Zhao X., Yang C., Gu J., Weng W., Hui J., Mao Q., Gao G., Feng J. (2019). Risk factors of hospital mortality in chronic subdural hematoma: A retrospective analysis of 1117 patients, a single institute experience. J. Clin. Neurosci..

[18] Bartek J., Sjåvik K., Dhawan S., Sagberg L.M., Kristiansson H., Ståhl F., Förander P., Chen C.C., Jakola A.S. (2019). Clinical Course in Chronic Subdural Hematoma Patients Aged 18–49 Compared to Patients 50 Years and Above: A Multicenter Study and Meta-Analysis. Front. Neurol..

[19] Abe Y., Maruyama K., Yokoya S., Noguchi A., Sato E., Nagane M., Shiokawa Y. (2017). Outcomes of chronic subdural hematoma with preexisting comorbidities causing disturbed consciousness. J. Neurosurg..

[20] Link T.W., Schwarz J.T., Paine S.M., Kamel H., Knopman J. (2018). Middle Meningeal Artery Embolization for Recurrent Chronic Subdural Hematoma: A Case Series. World Neurosurg..

[21] Hanif H., Farook S., Suriya S.S., Gondal M.U.R., Bilal M.I., Sheikh A.B. (2022). Middle Meningeal Artery Embolization: A Paradigm Shift in Approach of Chronic Subdural Hematoma. J. Community Hosp. Intern. Med. Perspect..

[22] Oishi M., Toyama M., Tamatani S., Kitazawa T., Saito M. (2001). Clinical Factors of Recurrent Chronic Subdural Hematoma. Neurol. Med. Chir..

